# Genetically Engineered T-Cells for Malignant Glioma: Overcoming the Barriers to Effective Immunotherapy

**DOI:** 10.3389/fimmu.2018.03062

**Published:** 2019-01-22

**Authors:** Pavlina Chuntova, Kira M. Downey, Bindu Hegde, Neil D. Almeida, Hideho Okada

**Affiliations:** ^1^Department of Neurological Surgery, University of California, San Francisco, San Francisco, CA, United States; ^2^George Washington University School of Medicine and Health Sciences, Washington, DC, United States; ^3^The Parker Institute for Cancer Immunotherapy, University of California, San Francisco, San Francisco, CA, United States; ^4^Cancer Immunotherapy Program, University of California, San Francisco, San Francisco, CA, United States

**Keywords:** T lymphocyte, brain cancer, Glioblastoma, TCR - T cell receptor, CAR (chimeric antigen receptor) T cells, Glioma

## Abstract

Malignant gliomas carry a dismal prognosis. Conventional treatment using chemo- and radiotherapy has limited efficacy with adverse events. Therapy with genetically engineered T-cells, such as chimeric antigen receptor (CAR) T-cells, may represent a promising approach to improve patient outcomes owing to their potential ability to attack highly infiltrative tumors in a tumor-specific manner and possible persistence of the adaptive immune response. However, the unique anatomical features of the brain and susceptibility of this organ to irreversible tissue damage have made immunotherapy especially challenging in the setting of glioma. With safety concerns in mind, multiple teams have initiated clinical trials using CAR T-cells in glioma patients. The valuable lessons learnt from those trials highlight critical areas for further improvement: tackling the issues of the antigen presentation and T-cell homing in the brain, immunosuppression in the glioma microenvironment, antigen heterogeneity and off-tumor toxicity, and the adaptation of existing clinical therapies to reflect the intricacies of immune response in the brain. This review summarizes the up-to-date clinical outcomes of CAR T-cell clinical trials in glioma patients and examines the most pressing hurdles limiting the efficacy of these therapies. Furthermore, this review uses these hurdles as a framework upon which to evaluate cutting-edge pre-clinical strategies aiming to overcome those barriers.

## Introduction

Malignant gliomas, including glioblastoma (GBM), are the most common form of malignant primary brain tumors. Among those, GBM represents the most common and aggressive tumors with an average survival rate of 15 months following diagnosis (1). The current standard of care involves maximal safe tumor resection followed by radiotherapy and chemotherapy. Despite advances in cytotoxic therapy regimens, targeted angiogenesis inhibitors and novel therapeutic modalities, such as alternating electric field therapy, patient survival has only improved modestly over recent years (2). GBM may occur *de novo* in multiple types of neuro-epithelial cells, which is diagnosed as primary GBM, or it may arise following the progression or recurrence of low-grade glioma (LGG) into high grade form (HGG), in which case it is diagnosed as secondary GBM. Primary GBM is more prevalent, confers worse prognosis, and is understood to develop from distinct genetic precursors compared to secondary GBM (3). In addition to the distinction between primary and secondary GBM, malignant gliomas represent the most common mortality and morbidity among pediatric cancers. Especially, high grade gliomas that affect the midline structure of the brain [diffuse midline gliomas (DMG)] are among the poorest responders to existing treatments, due in part to the unique genetic and epigenetic mechanisms driving the development of these tumors (4). The wide differences in tumor etiology and genetic landscape among GBM necessitate different treatment approaches and have resulted in a patient population with an acute need for improved therapy.

The central nervous system (CNS) was once considered an immune privileged site that was spared from the potentially damaging effects of active immune responses (5, 6). However, decades of research into the role of the immune system within the CNS has amended this preconception and allowed for a deeper understanding of how the adaptive immune response can function in the CNS [reviewed in (7)]. Recent studies investigating peptide vaccines and adoptive cell transfer for patients with malignant glioma have demonstrated that systemically administered treatments can, in fact, elicit antigen-specific T-cell responses. Despite these encouraging data, however, therapeutic responses were observed infrequently and had variable durations (8–12). The results of these initial trials underscore the need for continued in-depth research and analysis of the immunotherapeutic approaches for the treatment of glioma patients.

The successes of chimeric antigen receptor (CAR) T-cell therapy in hematological cancers have renewed the hope that durable remissions may become possible for patients with solid cancers, including those with GBM. Brain tumor patients have proven to be a particularly challenging population to treat with immunotherapy as many of the characteristics of a productive immune response, such as edema and widespread inflammatory infiltration, can have a devastating effect when they occur within close proximity to neural tissues. Despite these increased risks, genetically engineered T-cells, such as CAR T-cells, have the potential to improve the survival outcomes for patients. Tumor-targeting CARs are genetically engineered receptors that combine the antigen specificity of antibodies through the use of single chain variable fragments (scFv) with the potent antitumor effects of activated T-cells (13). However, the use of antibody-derived scFv limits antigen selection to surface bound proteins. Therefore, multiple groups, including ours, have begun to evaluate genetically engineered T-cells expressing a physiological form of tumor antigen-reactive T-cell receptor (TCR) in patients where tumor-specific neoantigens are derived from intracellular proteins (14). Regardless of the mode of antigen recognition, genetically engineered T-cell therapy in brain tumor patients has encountered a panoply of challenges. Some of these hurdles may be shared among all solid tumor types, such as antigen heterogeneity and tumor-derived immunosuppression, while other challenges are characteristic to CNS malignancies, such as the absence of professional antigen-presenting cells and the limitations to lymphocyte homing resulting from the blood-brain barrier.

In this review, we will highlight the most recent clinical status of CAR T-cell therapy for malignant glioma and then discuss the major challenges facing CAR T-cell immunotherapy in GBM, including neuroanatomical considerations, barriers to effector T-cell trafficking, immunosuppression in the GBM microenvironment, antigen heterogeneity, off-tumor toxicity, as well as the diverse challenges and opportunities afforded by concomitant therapies in the clinic. Furthermore, we will use these challenges as a framework to evaluate strategies for engineering more effective and specific CAR T-cell therapies for glioma.

## Clinical Experiences With GBM CAR T-Cell Therapy

The clinical utility of CAR T-cells targeting CD19 in relapsed and refractory B cell malignancies has proven to be exceptional in these patient populations (15, 16). However, the efficacy of CAR-T therapy in solid tumors has been less evident (17). Despite the complex barriers associated with treating CNS cancers, several early phase CAR T-cell clinical studies provide encouraging data.

### GBM-Specific CAR T-Cell Targets

GBM are generally considered to be immunologically cold tumors due in part to the overall low mutation loads of these tumor cells (18). One of the key challenges that has impeded development of CAR therapies for GBM is the limited availability of targetable tumor-specific antigens which do not confer any risk of toxicity toward normal tissues. An attractive mutation resulting in the formation of a common neoantigen in the GBM context is variant III of the epidermal growth factor receptor (EGFRvIII). This truncated receptor is expressed in 20% of newly diagnosed GBM patients and has not been found to be expressed on normal tissues, rendering it tumor-specific (19–21). It is characterized by an in-frame deletion of exons 2–7, which confers ligand-independent constitutive signaling through EGFR that results in cellular proliferation and enhanced resistance to both radio- and chemotherapies. The generation of a glycine at the splice-junction between exons 1-8 provides a surface epitope that can be readily targeted by immunotherapeutic approaches (21).

In a phase I clinical trial, O'Rourke and colleagues treated 10 recurrent GBM patients with a single intravenous infusion of autologous EGFRvIII-specific CAR T-cells. The group observed no objective radiographic response, apart from one patient who presented with stable residual disease for over 18 months. The patients did not suffer any off-tumor toxicities or cytokine release syndrome, providing evidence that systemic infusion of EGFRvIII-CAR T-cells is feasible and safe (12). Importantly, the authors observed significant but transient expansion of the CAR T-cells during the course of treatment and successful infiltration of CAR T-cells in the tumor site, which was ultimately associated with the decrease of EGFRvIII-expressing tumor cells. In addition, the research team noted increased and robust upregulation of several immune inhibitory molecules, such as programmed death ligand receptor-1 (PD-L1) and indoleamine-2,3-deoxygenase 1 (IDO1). The presence of CAR T-cells at the tumor site is evidence that systemically infused T-cells can be activated and recruited to the brain. While these observations are encouraging, the failure of this therapy to achieve objective clinical responses underscores the potentially debilitating impact of antigen heterogeneity and local immune suppression on CAR therapy which often manifests in the outgrowth of antigen loss variants.

### GBM-Associated CAR T-Cell Targets

IL-13 receptor α2 (IL-13Rα2) is a promising non-mutant GBM-associated antigen due to its broad tumor expression and extremely low expression levels in normal brain (22). This monomeric high affinity receptor binds IL-13 but not IL-4 and drives the production of transforming growth factor-β (TGF-β) in the tumor microenvironment (TME) (23). IL-13Rα2 is overexpressed in 75% of GBM patients and is a prognostic indicator for poor patient survival (24). Initial studies by Brown et al. evaluated the effect of repeated intracranial injections of IL13Rα2-targeting CD8^+^ CAR T-cells in 3 patients with recurrent GBM (25). The treatment was well-tolerated and resulted in transient antitumor activity in two of three patients. However, the authors noted that residual tumor tissue adjacent to the site of injection displayed significantly lower expression of IL13Rα2, implying antigen loss as a result of therapy. The same group subsequently reported a case study where they observed regression of an IL13Rα2-positive multifocal GBM tumor in a patient treated with intraventricular administrations of second generation IL13Rα2-CAR T-cells that also express CD137 intracellular domain as part of the CAR construct (26). The authors observed transient complete response of all cranial and metastatic tumors after repeated infusions. However, the patient eventually succumbed to metastatic recurrent lesions with decreased expression of IL13Rα2, highlighting the importance of developing improved strategies for overcoming acquired immune resistance on a systemic scale.

Another Phase I clinical trial by Ahmed et al. targeting the tumor-associated antigen human epidermal growth factor receptor-2 (HER2) reported the outcome of treating 17 GBM patients with HER2-specific CAR T-cells (27). The authors reported no serious adverse events following the administration of dose-escalating treatments and the observation of clinical benefit in 8 of 17 patients (1 partial response and 7 stable disease). The autologous T-cells used to manufacture CAR T-cells in this study were selected to be virus-specific. Because 16 of the 17 patients tested seropositive for cytomegalovirus, the investigators hypothesized that expression of the CAR construct in virus-specific CD8^+^ T-cells would optimize the persistence of CAR T-cells if the T-cells were to receive survival and proliferation signals via their endogenous TCR. Unfortunately, the CAR T-cells did not expand and persisted in only low levels in the periphery, suggesting the need to further develop methods of enhancing CAR T-cell survival and expansion *in vivo*.

While the positive safety profiles reported by all four studies are encouraging, these data highlight the substantial challenges facing CAR T-cell therapy for GBM. One key finding from all three recently completed Phase I studies was the low level expansion and persistence of the infused CAR-T-cells. Variable expansion and trafficking of T-cells to the brain tumor site, the dynamic immunosuppressive response mounted by the TME, and antigen loss in post-therapy recurrent tumors may explain some of this lack of expansion and persistence. We will start by investigating each of these sets of challenges in more detail and then review the strategies currently being explored to address them in the setting of malignant glioma.

## Neuroanatomical Challenges and T-Cell Homing

The efficacy of immunotherapy for malignant glioma relies upon the ability of therapeutic immune cells to reach the brain parenchyma and induce an anti-tumor response. Although adaptive immunity plays a critical role in immune surveillance of the CNS, the CNS has developed mechanisms that tightly regulate entry and activation of innate and adaptive immune cells to limit the potential side effects of neuroinflammation. It is important to recognize that the effects of inflammation, such as edema, cytokine-induced toxicity, and neurodegeneration, can be detrimental to the functional integrity of the CNS. Understanding of the neuroanatomical features that underlie these mechanisms is essential for the successful development and application of genetically engineered T-cells for malignant glioma. The CNS was historically considered a site of immune privilege because neither allografts transplanted in the brain of immune- competent mice nor the inoculation of viral and bacterial pathogens into the brain parenchyma elicited immunological responses (5, 6, 28, 29). These findings were initially attributed to the presence of the BBB, absence of lymphatics, and the relative incompetence of antigen presenting cells in the CNS. However, several decades of research into neuro-inflammatory conditions and clinical oncology have challenged these notions [reviewed in (7)]. It is currently understood that the CNS is neither completely privileged from systemic immunity nor impermeable to activated immune cells (9, 30, 31). Nevertheless, the unique anatomical features of the CNS pose several challenges that impede the ability of T-cells to recognize and respond to antigens within the brain. This section discusses these features and outlines a variety of strategies to overcome these impediments.

### Anatomical Considerations of the Immune Response

The CNS can be broadly divided by the areas which are protected by the BBB and those that are not, which has important consequences for the efferent arm of the immune response. The ventricles, meninges, and spinal cord are not protected by the BBB and are bathed in the cerebrospinal fluid (CSF) produced by the choroid plexus (32). The brain parenchyma and its interstitial fluid (ISF) are anatomically separated from both the peripheral bloodstream and the CSF by the BBB. The brain parenchyma lacks conventional lymphatic vessels and instead relies upon the drainage of tumor antigens and immune cells through the ISF and CSF into the dural, cervical, and nasal lymphatics. Access to these peripheral lymphatics depends upon anatomical location within the CNS. Both the cellular and soluble components of the CSF in the ventricular and subdural spaces can drain efficiently to the peripheral lymphatics (33–35). However, these same components within the ISF of the brain parenchyma are anatomically restricted from reaching the peripheral lymphatic system. Instead, the parenchyma must rely upon the limited exchange of CSF and ISF, termed the glymphatic system, in order for soluble antigens and signaling molecules to reach the peripheral lymphatics (36, 37). The absence of conventional lymphatic access to the parenchyma greatly hinders the afferent arm of the adaptive immune system needed for antigen presentation and the initiation of a systemic immune response to a tumor.

**Figure 1:**
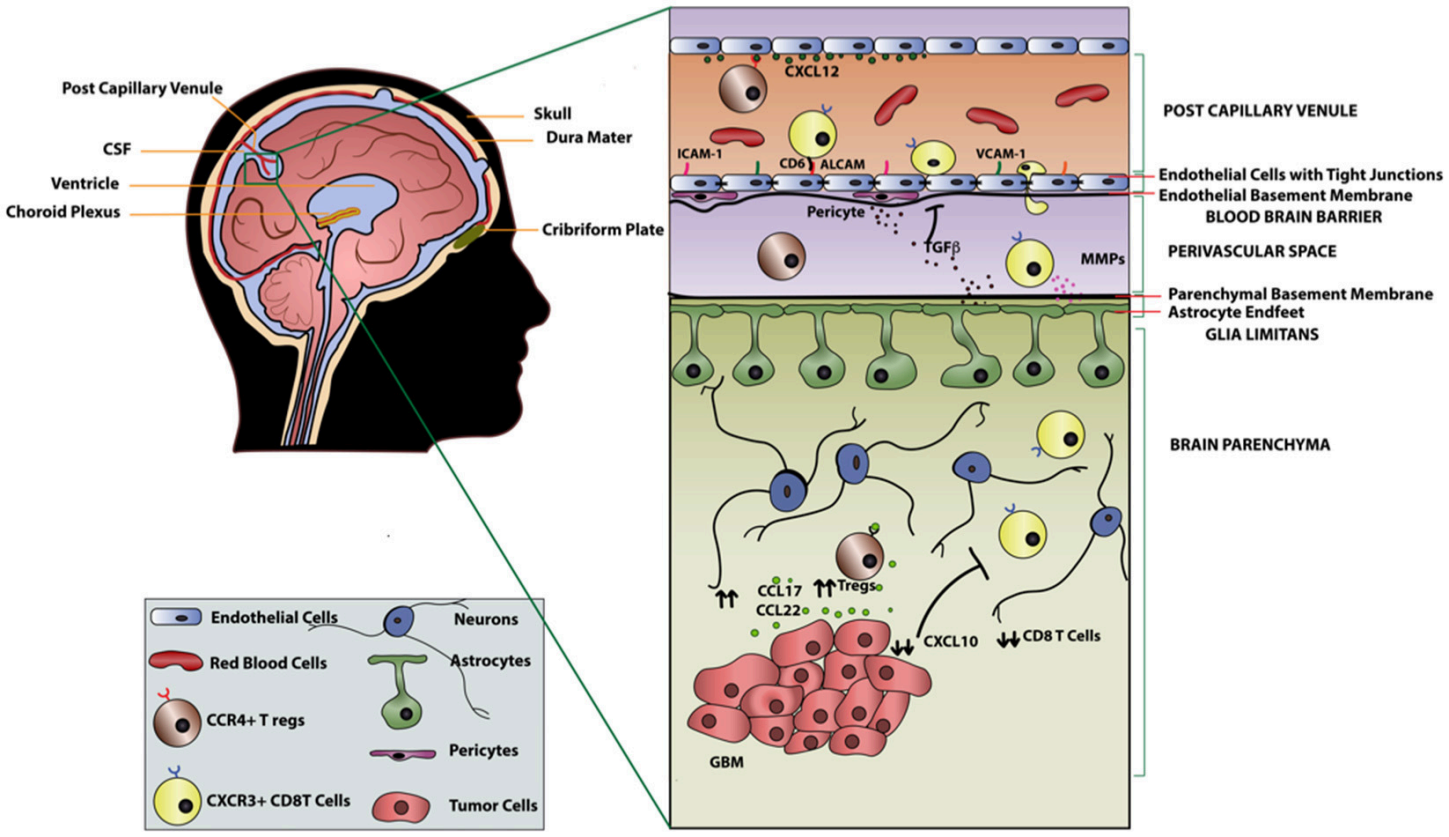
T-cell migration across the blood brain barrier in GBM.

The BBB is a permeability barrier composed of tight junctions connecting endothelial cells with the luminal and abluminal membranes lining the capillaries of the brain (38). Although not an absolute barrier, the BBB restricts the entry of ionic substances, large molecules, and naïve immune cells from the peripheral blood into the brain parenchyma. Lymphocyte entry into the brain parenchyma is tightly regulated (Figure 1) by the BBB as well as the glia limitans, which is formed by the fusion of astrocyte processes lining the parenchymal basal membrane along the entirety of the CNS (39). The BBB selectively allows activated but not naïve T-cells to enter the brain (40–42). Therefore, in the absence of inflammation, the brain parenchyma is largely devoid of immune cells. However, it is important to recognize that T-cells can cross the BBB and infiltrate the brain parenchyma given the right circumstances (43–45).

### T-Cell Recruitment to the Brain Parenchyma

Although the infiltration of immune cells is heavily restricted, there are a few mechanisms by which a small number of lymphocytes and antigen presenting cells can enter the CNS: (i) via the post-capillary venules into the perivascular space; ii) by extravasation through the choroid plexus of the ventricles into the CSF; or iii) through superficial leptomeningeal vessels into the subarachnoid space (46, 47). We will discuss the first mechanism in detail as it pertains most directly to the recruitment of T-cells into the brain parenchyma.

Recruitment of T-cells into the brain parenchyma is a sequential, coordinated process beginning with the binding of integrins α4β1 and lymphocyte associated antigen-1 (LFA-1) expressed on activated T-cells to the adhesion molecules vascular cell adhesion molecule 1 (VCAM1) and intracellular cell adhesion molecule 1 (ICAM1) on endothelial cells, respectively (39). Adhesion of cells to endothelial cells of the CNS also involves a tissue-restricted adhesion molecule, activated leukocyte adhesion molecule (ALCAM) which binds CD6 on mature T-cells (48). The rolling of T-cells established by these ligand-binding interactions leads to the activation of G protein-coupled receptors on the T-cells, resulting in conformational changes that promote tight binding of integrins to cell adhesion molecules on the endothelium. Following these integrin-adhesion molecule interactions, T-cells traverse through the endothelial lining and reach the perivascular space. Activated T-cells must then cross the glia limitans to enter the brain parenchyma. The entry of T-cells into the brain parenchyma is regulated by matrix metalloproteases (MMPs) secreted by other T-cells (49). Furthermore, factors such as tumor necrosis factor-α (TNFα), IL-12, TGFβ, and IL-6 secreted by astrocytes of the glia limitans in an inflammatory setting additionally regulate entry of activated T-cells across the BBB (50, 51). Similarly, increased expression of cell adhesion molecules in malignant glioma as well as neuro-inflammatory conditions, such as such as multiple sclerosis (MS) and experimental autoimmune encephalomyelitis, was shown to increase infiltration of T-cells into the brain parenchyma (48, 52, 53). Another critical factor that dictates T-cell recruitment into the parenchyma is antigen-specificity. Galea et al. demonstrated that antigen specific CD8^+^ T-cells can traffic to the site of the brain where cognate antigen is present, while CD4^+^ T-cells can traffic across the BBB regardless of antigen specificity (38).

Interestingly, it is clear from radiographic imaging of GBM that these tumors regularly disrupt the BBB to an extent that varies within tumors and between patients (54). In particular, glioma cells have been shown to breach the BBB by decoupling vasculature from the astrocytic endfeet that maintain BBB integrity and potentially increase exposure of the tumor to administered therapeutics (55). Nevertheless, BBB is intact in portions where glioma cells infiltrate into the normal brain tissue, and thus novel strategies will be necessary to overcome the tight regulation of lymphocyte trafficking into the brain parenchyma for the success of immunotherapy. From our discussion of CNS anatomy and T-cell recruitment, it is clear that the mechanisms by which T-cells enter the brain parenchyma are complex and require multifaceted considerations of the broader circumstances in the CNS environment. A deeper understanding of the mechanisms underlying T-cell recruitment, especially as it pertains to the heterogeneous settings of malignant glioma, will be required for the development of safe and effective genetically engineered T-cell therapies.

### Strategies to Overcome the Unique Neuroanatomical Challenges of the Brain

#### Regional Delivery

**Figure 2:**
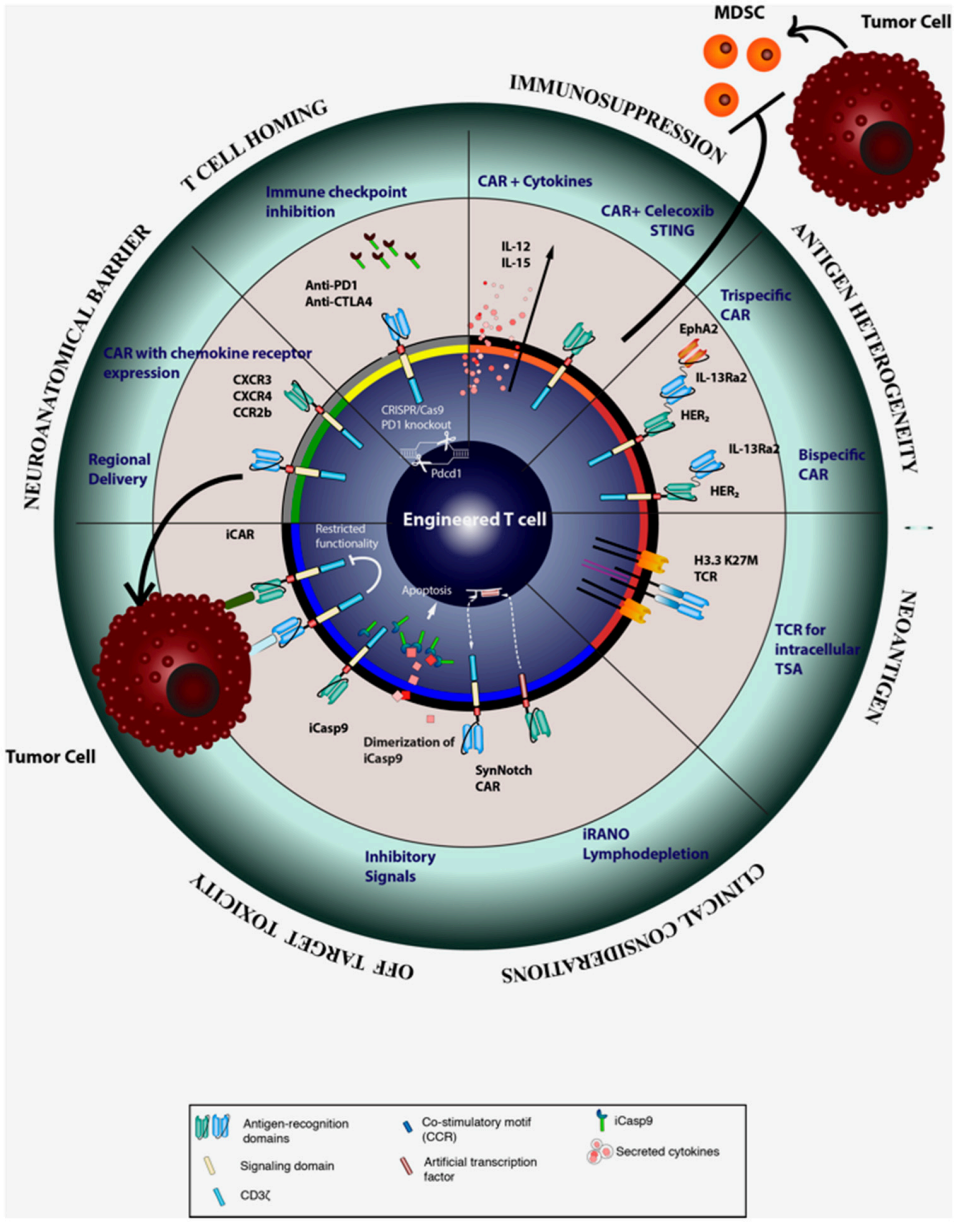
Strategies for improving the efficacy CAR T cell therapy.

To circumvent the difficulties of CAR T-cell trafficking into the brain parenchyma and to reduce systemic toxicity associated with intravenous delivery, several investigators have initiated clinical trials to study the safety and efficacy of regional delivery of CAR T-cells (Figure 2). Regional delivery has been attempted to improve CAR T-cell localization in ovarian cancer, mesothelioma, lung cancer, breast cancer, and squamous cell cancer of the head and neck (NCT02498912, NCT02414269, NCT01818323). Several authors have established the safety and efficacy of intracranial or intrathecal delivery of EGFRvIII and IL13Rα2 CAR T-cells in preclinical models of GBM. Currently, there have been three clinical trials using regional delivery of CAR T- cells as an approach to compensate for poor T-cell homing and reduce systemic toxicity (25, 26, 56, 57). Yaghoubi et al. treated a GBM patient via intracranial delivery of IL12Rα2-specific CAR T-cells after resection of initial tumor. Tumor regression was observed and T-cells persisted for more than 5 weeks without adverse effects (56). As discussed prior, Brown and colleagues have conducted two clinical trials exploring the local administration of IL-13Rα2 CAR T-cells into GBM patients. In one patient with multiple lesions with meningeal disseminations, who received repeated intraventricular administration of IL-13Rα2 CAR T-cells, persistence of the CAR T-cells was seen in the CSF for at least 7 days after the last intracranial infusion, and the patient had a complete response for 7 months before the tumor recurred. The authors also observed a robust increase in inflammatory cytokine and chemokine induction in the CSF after infusion compared to the baseline levels without observable increase in the peripheral blood (26). Similarly, another ongoing clinical trial providing autologous peripheral blood mononuclear cells transduced with EGFRvIII CAR directly into the tumor site aims to increase the efficacy of CAR therapy and reduce the systemic off-site effects (NCT03283631). While promising, it is important to recognize that intra-CSF delivery of CAR T-cells does not necessarily mean effective delivery to the brain parenchyma, where most glioma tissues reside. Furthermore, the post-infusion persistence of transferred T-cells remains to be elucidated as preclinical studies continue to show varied results (58). The lack of lymphoid organs in the brain to support lymphocyte survival may be one of the factors driving this diminished persistence.

#### CAR T-Cells Expressing Chemokine Receptors

Efficacy of systemic delivery approaches, such as intravenous infusion, depends on trafficking of CAR T-cells to the tumor site. In addition to adhesion molecules that we discussed earlier in this review, the ability of CAR T-cells to effectively localize to the tumor site also requires expression of chemokine receptors corresponding to chemokine ligands expressed by the tumor (59). Amankulor et al. reported that the T-cell attracting chemokines CXCL9, CXCL10, CCL2, and CCL12 are downregulated in *isocitrate dehydrogenase (IDH)*-mutated gliomas, resulting in the repression of immune cell infiltration (60). On the other hand, CCL17 and CCL22, which promote recruitment of CCR4^+^ T regulatory cells (Tregs), are upregulated in GBM (61). Interestingly, the expression of CCL2 by some gliomas, which attracts CD8^+^ T-cells, has been exploited by investigators for adoptive T-cell strategies (62, 63). Another way of improving homing of CAR T-cells to the tumor site is by engineering CAR T-cells that co-express chemokine receptors (Figure 2). Our group and others have found that CXCR3, along with its ligands CXCL9 and CXCL10, plays predominant roles in cytotoxic lymphocyte trafficking into the glioma tumor site (45, 64, 65). We have also shown that adjuvant polyinosinic-polycytidylic acid stabilized with polylysine and carboxymethylcellulose (poly-ICLC) provided systemically can promote cytotoxic lymphocyte trafficking into gliomas in an IFN-α and IFN-γ dependent manner through induction of CXCL10 (44).

Additionally, expression of CXCL12 and its receptors, CXCR4 and CXCR7, in the CNS plays important roles in determining whether lymphocytes can gain entry in the CNS under normal and inflammatory conditions. Polarized expression of CXCL12 on the basolateral surface of endothelial cells of the BBB retains CXCR4 expressing leukocytes in the perivascular space and prevents extravasation into the CNS parenchyma under normal conditions (66). During pathological conditions such as multiple sclerosis, polarized expression of CXCL12 is disrupted by overexpression on the luminal side of the endothelium, thereby resulting in enhanced leukocyte trafficking into the CNS. Klein and colleagues have demonstrated that blockade of CXCR4 on T-cells could facilitate lymphocyte escape from the perivascular space into the CNS parenchyma (67). Additionally, both CXCR4 and CXCR7are highly overexpressed in patient-derived glioma cells (68, 69) and play a critical role in progression of the disease (70). While CXCR4 antagonism inhibited GBM tumor growth in various pre-clinical models (71–73), its direct role in recruitment of T-cells in GBM is yet to be determined.

#### Focused Ultrasound

In addition to strategies intended to enhance homing to the tumor site, there are therapies aimed at disrupting the BBB that have yet to be tested in the setting of CAR T-cells. High intensity focused ultrasound (HIFU) is a thermal ablation technique that has shown to increase activated TIL migration into solid tumors including breast, liver, pancreas, kidney and bone cancer (74–76). While HIFU is shown to disrupt the BBB, it is also accompanied by some tissue damage (77). Therefore, an alternative approach entitled Focused Ultrasound (FUS), which uses intensities similar to diagnostic ultrasounds, is used along with microbubbles injected intravenously for regional delivery of drugs and cytokines into the brain parenchyma (78). Chen et al. observed an increase in tumor infiltrating lymphocytes and cytotoxic lymphocytes, in particular, after FUS exposure in the presence of microbubbles (79). Although promising, the possibility of using FUS to increase localization of CAR T-cells to the brain safely needs to be thoroughly researched.

## Immunosuppression in the Glioma Microenvironment

### Tumor Cell Intrinsic Mechanisms

The genomic landscape of glioma is complex and encompasses structural rearrangements, mutations in signature oncogenes (*EGFR, TP53*, etc.), as well as chromosome modifying proteins such as *ATRX* and *IDH* (19). Among patients with LGG or secondary GBM, mutations within *IDH1* and *IDH2* have been reported in 70–80% of cases (80). The single amino acid change within the isocitrate-binding domain (R132 in IDH1; R140 or R172 in IDH2) confers a gain-of-function mutation leading to the accumulation of the oncometabolite 2-hydoxyglutarate and potential genome-wide epigenetic changes. Our group recently reported that *IDH*-mutant glioma cells are able to influence the tumor immune environment through the suppression of type 1 immune response genes (81). We observed decreased overall expression and activation of signal transducer and activator of transcription 1 (STAT1) and significantly lower levels of the effector T-cell attracting chemokines, such as CXCL10, produced by IDH-mutant glioma cells. Furthermore, a study by Berghoff and colleagues reported a significantly lower rate of T-cell infiltration in *IDH*-mutant vs. *IDH*-wildtype gliomas (82). These studies provide evidence that genetic alterations intrinsic to the glioma tumor cells are able to alter the cellular composition of the TME and aid in immune evasion. Therefore, novel immunotherapeutic approaches need to address the downstream consequences of tumor cell intrinsic mutations in addition to targeting tumor antigens.

In addition to genetic mutations, GBM cells display a vast array of molecular signaling alterations, such as the increased expression and activation of STAT3 (83). Activation of STAT3 results in dynamic transcriptional changes depending on the cellular context (84). In GBM, phosphorylated STAT3 (p-STAT3) has emerged as a major regulator of immune suppression (85). Treatment of GBM patient-derived myeloid cells with the p-STAT3 small molecule inhibitor WP1066 resulted in upregulation of the co-stimulatory molecules CD80 and CD86. Furthermore, in the presence of WP1066, normally unresponsive patient T-cells were shown to proliferate when stimulated with autologous APCs (85). In addition to the immunosuppressive effects of STAT3 in immune cells, work by Wei and colleagues demonstrated that GBM-initiating cells have a constitutively active STAT3 pathway, and that inhibition of STAT3 significantly diminished the ability of these cancer-initiating cells to suppress T-cell expansion and induce Treg recruitment (86). Based on these promising data, WP1066 is being evaluated in a phase I clinical trial for patients with recurrent GBM and melanoma patients with brain metastases (NCT01904123). Inhibition of STAT3 in the GBM microenvironment may significantly contribute to the efficacy of anti-GBM CAR T-cells, and thus the outcomes of this and any future STAT3-targeting clinical trials are highly anticipated.

The cellular arm of the immune system offers a potent, selective, and durable mechanism of protection through the tightly regulated interactions of T-cells and the vast array of peptides presented in the groove of human leukocyte antigen (HLA) molecules. Cytotoxic CD8^+^ T-cells depend on HLA class I- presented peptides for their activation. A major immune resistance mechanism in GBM is the downregulation of HLA class I expression on tumor cells (87). In certain cases, expression of HLA class I can be restored by treatment with IFN-γ; however, mutations leading to loss of heterozygosity (LOH) of the HLA class I and beta-2 microglobulin regions can result in irreversible downregulation of HLA class I. Our group has previously reported that 41% of analyzed GBM samples showed LOH in the HLA class I region, and this was significantly associated with shorter survival in newly diagnosed GBM patients (88). Downregulation of HLA class I expression can also be the result of changes to the cellular antigen-processing machinery which is involved in stabilizing and promoting the cell surface expression of HLA-I molecules. Tapasin is a protein known to facilitate the binding of peptides to class I molecules, and in its absence, the expression of HLA class I is significantly reduced (89, 90). Thuring et al. reported the significant correlation between tapasin and both HLA-I expression and GBM patients survival time (91). While CAR constructs must target surface antigens, a majority of cancer-specific neoantigens are derived from intracellular proteins. This certainly gives an advantage for TCR-based approaches. However, success of TCR-based approaches will likely require additional therapeutic strategies to ensure sufficient HLA expression levels in the tumor site.

### Secreted Factors in the Tumor Microenvironment

In addition to tumor cell intrinsic factors, various other mechanisms have been described that render the GBM microenvironment exceptionally immunosuppressive. These include the recruitment of Tregs and suppressive myeloid cells as well as the upregulation of immune checkpoint molecules and immunosuppressive cytokines [reviewed in detail (92)]. One approach proposed to counteract the immunosuppressive microenvironment is the co-expression of cytokines such as IL-12 and IL-15 by CAR T-cells (93, 94). IL-12 has been shown to enhance CD8^+^ T-cell activation and to act on surrounding innate immune cells by providing a type I differentiation signal. As a result, pre-clinical models suggest that tumor antigen-specific T-cells engineered to express IL-12, survive longer in the tumor milieu and are more effective at tumor clearance than CAR transgenic T-cells alone (93) As lack of CAR-T cell persistence *in vivo* is another recurring obstacle in both pre-clinical and clinical studies, researchers have engineered CAR-T cells to express the pro-T cell survival cytokine IL-15 in an activation-dependent manner. Krenciute and colleagues demonstrated that upon recognition of their cognate antigen, T-cells transduced with IL13Rα2-CAR and IL-15 upregulated production of IL-15 which enhanced the cells' effector function and their antiglioma activity *in vitro* and *in vivo* (94).

In addition to utilizing a double transgene strategy, the authors make use of an anti-IL13Rα2 CAR integrating an antibody-derived scFv as opposed to the zetakine-based IL13Rα2-CAR. This scFv-based CAR construct has been shown to exhibit improved antigen specificity as scFv-based CAR-T cells were able to recognize and kill IL13Rα2-expressing but not IL13Rα1-expressing target cells (95). Despite these therapeutic alterations, the authors reported that gliomas recurred in their xenograft model displaying lower expression of IL13Rα2, signifying the critical need that CAR-T cells promote immune responses against multiple tumor antigens.

Another hurdle to be overcome by adoptively transferred CAR T-cells is the high local levels of TGF-β in the TME of GBM (96). Introducing the dominant negative TGF-β type II receptor in addition to the CAR construct when manufacturing CAR T-cells renders them resistant to the effects of TGF-β and has been shown to enhance antitumor activity of the T-cells (97, 98). Furthermore, TGF-β inhibitors and blocking antibodies have been studied extensively pre-clinically. However, their therapeutic efficacy in glioma patients remains unconvincing, likely due to low BBB penetrance, underscoring the possibility that targeting TGF-β alone might not be sufficient to meaningfully impact disease progression (99, 100).

### Immunosuppressive Myeloid Cells

Myeloid cells constitute the largest subset of glioma immune infiltrates and can account for up to 50% of the total tumor mass (101, 102). A particular subset of these cells are known as myeloid derived suppressor cells (MDSC) and are generally recognized as a heterogenous population of immature myeloid cells able to support *de novo* gliomagenesis and produce pro-tumorigenic factors within already established tumors (103). Numerous strategies for MDSC depletion and inhibition have been developed, such as the use of the non-steroidal anti-inflammatory drug (NSAID) celecoxib or the administration of STING (stimulator of IFN genes) agonists (Figure 2). Our group reported that celecoxib inhibits the production of prostaglandin E2 thus inhibiting the accumulation of MDSCs in the tumor microenvironment (104). The use of celecoxib alone was able to enhance expression of CXCL10 and increase recruitment of cytotoxic lymphocytes to the tumors in a pre-clinical glioma model. Additional experiments demonstrated that intratumoral administration of the STING agonist c-di-GMP was able to relieve the immunosuppressive effect of MDSC *in vivo* (105). As a result of enhanced production of type I cytokines and chemokines, this treatment increased T-cell migration to the tumor site and improved overall survival of tumor-bearing mice. Therefore, the addition of these and other MDSC modulating strategies to traditional adoptive T-cell therapies may provide substantial clinical benefit.

### Enhancing CAR T-Cell Function With Immune Checkpoint Inhibitors

Finally, as discussed earlier in this review, both therapeutically administered as well as endogenously activated T-cells are subject to elevated levels of immune checkpoint inhibition in the tumor microenvironment. Recent studies have suggested that tumor-infiltrating lymphocytes within GBM have an increased expression of immune checkpoint molecules such as PD-1, CTLA-4, LAG3, and TIM-3 (27, 106). Similarly, CAR T-cells have also been observed to express immune checkpoint molecules and acquire an exhausted phenotype (107, 108). Upregulation of molecules such as CTLA-4 and PD-1 on T-cells is a natural consequence of T-cell activation and serves the purpose of preventing rampant immune cell reactivity (109). Solid tumors have been shown to co-opt this immune balance mechanism to suppress the local activation and proliferation of T-cells. The ligand for PD-1, PD-L1, is present on both tumor cells and infiltrating myeloid cells. Although a recent study by Nduom et al. reported a median PD-L1 expression of 2.8% in their study of 94 GBM samples, robust induction of PD-L1, which is presumably due to local IFN-γ production, on GBM tissues was observed in the recent EGFRvIII-CAR clinical trial (12, 110).

Blockage of CTLA-4 and PD-1 in murine solid tumor models has led to an increased expression of activation markers by T-cells, such as IFN-γ, IL-2, perforin, and granzyme; furthermore, these treatments have resulted in improved trafficking of activated T-cells to the tumor site (111, 112). While the FDA-approved checkpoint inhibitors are administered systemically, specific blockade of checkpoint molecules within the therapeutic T-cells would mitigate systemic toxicities. Cherkassky et al. developed CAR-T-cells co-transduced with a dominant-negative PD-1 receptor lacking all the intracellular signaling domains (107). Using a pleural mesothelioma model, the authors reported that CAR-T cells expressing the dominant-negative PD-1 receptor controlled the tumor growth more efficiently than the control CAR-T cells, owing to their enhanced survival and ability to evade activation-induced exhaustion. Furthermore, the PD-L1-PD-1 signaling for immunosuppression may take place not only the surface of interacting cells, but may also be mediated by soluble PD-L1 in extracellular vesicles (EVs). A recent report by Ricklefs et al. suggests that GBM-derived EVs, such as exosomes and microvesicles inhibit human T-cell activation and proliferation. This effect correlated with the amount of PD-L1 carried by the EVs and was partially reversed through the use of an anti-PD-L1 antibody (113). Clinical efforts testing the effects of anti-PD-1 therapy alone in patients with recurrent GBM failed to show improved overall survival when compared with other agents (114) (NCT02017717). However, ongoing clinical trials are currently evaluating the use of CAR T-cells with built in CTLA-4 and PD-1 blockade, CAR T-cells in combination with anti-PD-1/PDL1 (115) (NCT03170141, NCT02706405), and the use of CRISPR/Cas9 to disrupt PD-1 in CAR T-cells (NCT03208556) with the aim of increasing CAR T-cells efficacy (Figure 2).

### CAR T-Cell Persistence and Antigen Specific Memory

As the addition of checkpoint inhibitors may not be enough to re-energize T-cells that are exhausted or drive the persistence of antigen specific memory T-cells to prevent GBM recurrence (116), new strategies are being explored to address these aims. A study by Sengupta and colleagues has investigated the use of a glycogen synthase kinase 3 (GSK3) inhibitor for improving expansion and persistence of the CAR T-cells. GSK3 is constitutively active in naïve T-cells and is inactivated briefly during clonal expansion of the activated T-cell (117). At peak expansion, GSK3 becomes active and results in clonal contraction and ultimately death of the activated T-cell (118, 119). The specific blockade of this protein with small molecule inhibitors results in T-cell expansion and the generation of memory T-cells (120, 121). Sengupta and colleagues reported that IL-13Rα2 CAR T-cells treated with a GSK3 inhibitor showed reduced exhaustion and increased expression of an effector memory phenotype (CD62L^lo^/CD45RO^hi^/CD127^+^) (122). Based on the demonstrated protective effects of GSK3 inhibition on activated T-cells, the authors of this study administered the IL-13Rα2 CAR T-cells and GSK3 inhibitor to mice bearing subcutaneous GBM xenografts and demonstrated that mice re-challenged with tumor after initial clearance did not develop new lesions. Furthermore, they identified CAR^+^ effector memory T-cells in the draining lymph nodes and spleens of these animals at 100 days following initial CAR administration.

## Antigen Heterogeneity, Antigen Escape, And Off-Tumor Toxicity

### Antigen Heterogeneity and Escape in GBM

In addition to being tumor-specific, ideal candidate tumor antigens must be expressed homogenously on the surface of a majority of tumor cells to mediate effective tumor killing. Antigen heterogeneity has been a universal barrier to effective CAR therapy across cancer types, including in the setting of CD19-CAR for leukemia and lymphoma (123). GBM is especially challenging in this regard, as clinical studies for all major tumor-specific and tumor-associated antigens to date have observed outgrowth of antigen loss variants due to substantial heterogeneity within tumors (12, 25–27). Because of its desirability as a tumor-specific antigen and extensive characterization, we will focus on EGFRvIII here as a prototypical example of antigen heterogeneity in GBM.

A variety of EGFRvIII CAR variations have been tested pre-clinically, with alterations in number as well as type of co-stimulatory domains and these studies have demonstrated effective and specific tumor lysis in murine and patient-derived tumor models (124–129). However, as discussed earlier, the first clinical study testing a second-generation EGFRvIII CAR failed to demonstrate efficacy and instead highlighted the strong adaptive capabilities of GBM cells to escape the surveillance of CAR T-cells by eliminating or altering antigen expression over time (12). Pre-clinical studies have also demonstrated that EGFRvIII seems to evade T-cell based targeting approaches due to the vast heterogeneity in its expression on tumor cells (130). Active amplification and rearrangement of EGFR can be found throughout GBM tumors, regardless of EGFRvIII status. Moreover, EGFRvIII-positive subpopulations may give rise to EGFRvIII negative clones which can subsequently re-express EGFRvIII after undergoing epigenetic modification. The survival of antigen loss variants and the relative ease of reacquiring EGFRvIII may contribute to the consistent recurrence of GBM tumors following EGFRvIII-CAR T-cell therapy. Separate mechanisms guided by the same principles may underlie antigen loss and tumor recurrence in the settings of other GBM CAR antigens.

An important barrier to the pre-clinical evaluation of antigen loss in EGFRvIII-CAR T-cell therapy has been the absence of EGFRvIII^+^ GBM patient-derived cell lines and syngeneic mouse models that effectively recapitulate the dynamics of EGFRvIII heterogeneity in patient tumors and allow for accurate prediction of long-term EGFRvIII-CAR T-cell therapy success in the clinic. Recently, several groups have sought to overcome this barrier by engineering novel cell lines and pre-clinical models to better reproduce the heterogeneous nature of EGFRvIII and some glioma-associated antigens (24, 131). Similar methodologies will need to be explored for modeling other candidate antigens undergoing pre-clinical evaluation for CAR T-cell therapy.

### Combinatorial Approaches Utilizing Tumor-Associated Antigens

Antigenic profiling of GBM has revealed a vast availability of tumor-associated antigens that may be targetable with immunotherapy (132), yet development of CARs specific for those novel antigens is hindered by safety concerns with regards to systemic and on-target off-tumor toxicity. Several tumor-associated antigen targets have been exploited for the development of CAR. Ephrin type A receptor 2 (EphA2), IL-13Ra2, and HER2 represent promising tumor associated antigens that have been targeted both pre-clinically and clinically using CAR T-cell therapy in the setting of GBM (23, 133, 134). Efficacy for monovalent CAR T-cells targeting each of these antigens has been established in pre-clinical models (135–138). Multiple groups have attempted to address the hurdle of antigen heterogeneity and escape in GBM by engineering combinatorial approaches that simultaneously target multiple GBM-restricted antigens at once (Figure 2). Hegde, Grada, and colleagues have developed a tandem CAR combining the recognition of IL-13Rα2 and HER2, based on mathematical modeling that predicted 90% tumor killing in GBM patients with this antigen combination (139, 140). This group went on to demonstrate superior efficacy *in vivo* for the tandem CAR (tanCAR) construct over bivalent CAR targeting the same antigens and observed that, in contrast to bivalent CAR, IFNγ and IL-2 secretion from tanCAR^+^ T-cells was higher than simply an additive effect of two monovalent CARs (141). Unfortunately, tumors did eventually recur in all groups after antigen clearance and tanCAR T-cells were shown to develop comparable increases over time in PD-1 and LAG3, although not TIM3. Based on the analysis of antigen variability across GBM patient cell lines, Bielamowicz and colleagues have since developed a tri-cistronic CAR transgene encompassing IL-13Rα2, EphA2, and HER2, which they called universal CAR (UCAR) (142). The authors reported increased cytolytic potential for UCAR^+^ T-cells over bivalent CAR^+^ T-cells, which was at least partially due to a smaller and more highly organized immune synapse. Although using the trivalent UCAR^+^ T-cells resulted in significantly increased survival, tumors did recur in some mice between 40 and 60 days after the first T-cell injection following loss of all three antigens. Repeated observation of antigen loss begs the question of how many antigens must be targeted at once for critical mass to occur and drive the complete remission of malignant glioma (141). Further combinations of tumor-associated and tumor-specific antigens remain to be developed for targeting GBM while preventing acquired immune resistance in the form of antigen loss.

### Mitigating Off-Tumor Toxicity

One of the most important risks associated with CAR T-cell therapy is on-target off-tumor toxicity, particularly in the case of T-cells targeting tumor-associated antigens. With the exception of EGFRvIII, all of the GBM antigens that are currently being evaluated clinically may be expressed at low-levels on normal tissues, which can result in substantial toxicity. The risk of on-target toxicity increases with affinity of the engineered T-cells to their antigen targets, as well as the potency of the T-cells and antigen expression level on normal tissues (143). In a trial of high dose HER2-CAR T-cell for metastatic colon cancer, one patient died of respiratory failure after low levels of HER2 were engaged on the lung epithelium, but subsequent studies using modified and lower affinity HER2-CAR T-cell have not led to any additional case reports which suggests that these modifications may improve safety (143, 144). A high avidity TCR engineered to target the melanoma associated antigen A3 (MAGE-A3) was tested in a Phase I clinical trial and despite showing strong antitumor effects in most patients, this treatment led to the death of three of the patients receiving the highest dose regimens. This TCR was known to recognize another MAGE-A family member, MAGE-A12, with 10-fold higher affinity. After the death of these patients, MAGE-A12 expression was subsequently found on a subset of neurons in these patients and control brains by histopathological examination (145); these findings underscore the need for stringent characterization of CAR binding and cross-reactivity in normal tissues. Neurotoxicity, characterized by endothelial activation and increased permeability of the BBB, is also a concern for CAR therapy as it was observed in a patient following CD19-CAR T-cell therapy (146). In a pre-clinical murine model, while CAR T-cells targeting GD2 demonstrated a marked efficacy in DIPG xenograft models, peritumoral neuroinflammation during the acute phase of antitumor activity resulted in hydrocephalus that was lethal in a fraction of animals (147). Furthermore, fatal encephalitis resulting from low-level antigen expression on the cerebellum was recently observed following GD2 ganglioside CAR T-cell therapy for neuroblastoma (148). Cytokine release syndrome (CRS) is also an important risk of CAR T-cell therapies that must be managed in the clinical setting. However, none of the existing published trials of CAR T-cells targeting GBM antigens have resulted in CRS or elevated peripheral cytokine levels. Management of off-tumor effects, neurotoxicity, and the potential of CRS remain essential considerations in the development of novel CAR T-cell therapy for human trials.

Several novel approaches have been generated for engineering CARs to limit off-tumor and systemic toxicities that might have promising applications in the context of GBM (Figure 2). One important method that has yet to be explored in CNS cancers is the introduction of a latent suicide switch such as inducible caspase-9 (iCASP9) enzyme, which can be used to direct T-cell apoptosis following the administration of a small-molecule drug (149). One benefit of this strategy is the ability to rapidly deplete administered T-cells to resolve cases of CRS and acute tissue toxicity in the clinical setting. In a clinical trial utilizing iCASP9^+^ alloreplete T-cells after stem cell transplantation where graft vs. host disease was detected, the administration of a small molecule homodimerizer eliminated 85–95% of circulating T-cells within 30 min (150). Employing such an approach in the CNS will require utilizing small molecule drugs with ample ability to cross the BBB where prevention of toxicity to normal brain tissue is warranted. Inhibitory CAR T-cells (iCARs), which target a tumor antigen but co-express an off-switch that is stimulated by a normal tissue-derived cognate antigen, has also been proposed to minimize allogenic CAR T-cell activation in the context of CD19-CAR (151). Roybal, Morsut, and colleagues have recently developed a novel system utilizing a synthetic Notch receptor whose activation drives the transcription of a second generation CAR (Syn-Notch CAR).The goal of this circuit is to prevent any CAR T-cell activation without the separate and sequential engagement of two cognate antigens which may be derived from either the tumor or the tissue microenvironment (152, 153). They reported that Syn-CAR^+^ T-cells failed to become activated in the absence of either antigen and demonstrated superior tumor-killing efficacy over bivalent CAR^+^ T-cells (153). An alternative strategy to mitigate off-tumor toxicity is a switch-mediated CAR that uses an antigen-specific antibody-based molecule which specifically binds the administered SwitchCAR^+^ T-cells. The binding of these antibody-based switches drives immunological synapse formation between SwtichCAR T-cells and tumor cells in a dose-dependent manner in xenograft models of CD19^+^ and CD20^+^ hematological malignancies, respectively (154, 155). This methodology has also been effective in targeting HER2^+^ breast cancer (156). Despite the plethora of methods being explored for CAR engineering for mitigation of on-target off-tumor and systemic toxicity, these strategies have yet to be evaluated in the context of GBM.

### Comparing Efficacy of CD4^+^ and CD8^+^ CAR T-Cell Subsets in Glioma

While most of the clinical trials we have discussed thus far have used a mixture of CD4^+^ and CD8^+^ T-cells (12, 26, 27) or CD8^+^ T-cells alone (25), there have been recent reports that CD4^+^ CAR T-cell subsets, in particular, may promote antitumor efficacy. Pre-clinical models, including a model of GBM, utilizing CD4^+^ cells transduced with a tumor-specific CAR have been found to aid in tumor-killing by other T-cell subsets as well as to lyse tumor cells directly (157, 158). In the setting of CAR T-cell therapy for solid tumors, the existence of CD4^+^ subsets has been found to increase CAR T-cell activity and persistence *in vivo* (58, 159). A recent pre-clinical study directly compared efficacy of a second generation IL-13Rα2 CAR transduced into patient-derived CD8^+^ or CD4^+^ T-cells and found CD4^+^ CAR T-cells demonstrated enhanced tumor killing and persistence compared with CD8^+^ and a mixed CD4^+^/CD8^+^ population in a xenograft model of GBM (159). The CD4^+^ CAR T-cells in this study secreted more IFNγ and IL-2 than CD8^+^ T-cells, while the CD8^+^ CAR T-cells more readily began expressing exhaustion markers.

### TCR Approaches for Targeting Malignant Glioma

Because CAR targets are limited to surface expressed antigens, the abundance of tumor-specific neoantigens derived from intracellular proteins has driven the development of TCR-based approaches (Figure 2). The histone H3 position 27 lysine to methionine substitution (H3.3 K27M) mutation is shared across 70% of diffuse intrinsic pontine glioma (DIPG) patients and a majority of DMG patients (160). It results in a global decrease of methylation at H3K27me3 and results in the suppression of polycomb repressive complex 2 (PRC2) and altered gene expression (161). Overall survival in DIPG patients with this mutation is shorter compared with patients harboring wild type H3.3 (160). We recently identified an HLA-A^*^02^*^:01-restricted epitope which includes the H3.3 K27M mutation, and we cloned cDNA of TCR α- and β-chains from this clone for transduction into T-cells. In this report, we showed that T-cells transduced with this TCR specific for H3.3K27M efficiently killed H3.3K27M^+^ glioma cells *in vitro* in an antigen- and HLA-specific manner (14). Furthermore, these TCR transduced T-cells also suppressed the progression of intracranial glioma xenografts in mice when used in adoptive transfer studies. These data are the basis for an upcoming Phase I clinical trial administering adoptively transferred T-cells with our transduced H3.3 K27M TCR. While the H3.3 K27M TCR has been effective in murine models of H3.3K27M^+^ malignant glioma, the effectiveness of TCR approaches in patients may require assurance of HLA class I expression, as discussed earlier. Nonetheless, this strategy remains a potent tool for targeting immunogenic epitopes which are not surface expressed but can be routinely presented by HLA Class I.

In order to develop effective TCR-based therapeutic approaches targeting antigenic heterogeneity of malignant glioma, additional novel tumor-specific neoantigens will need to be identified. A variety of deep sequencing and *in silico* HLA docking approaches have been employed with the aim of identifying neoantigens that can be effectively targeted by CAR and TCR approaches (162–164). These immunogenomics approaches are especially relevant in the context of GBM as 20–30% of recurrent GBM have been found to exhibit a hypermutator phenotype and may provide a rich supply of antigens for achieving complete patient response (162).

### Re-discovering Glioma Antigens for CAR T-Cell Therapy

In addition to EGFRvIII, EphA2, IL-13Ra2, and HER2, several other tumor-associated antigens have previously been explored as targets for GBM therapies in preclinical models (165–167). CD70 is found to be highly expressed in both primary and recurrent LGG and GBM, particularly in association with wild-type IDH expression (168). It has been shown to play an important role in recruiting immunosuppressive myeloid cells to the tumor microenvironment and CD70-CAR T-cells have demonstrated remarkable efficacy in patient xenograft and syngeneic murine tumor models (169). Chondroitin sulfate proteoglycan 4 (CSPG4) represents another emerging target for GBM CAR T-cells with high expression of this antigen in two-thirds of GBM patient specimens with little expression on normal tissues (170). GBM neurosphere engraftment in nude mice followed by the infusion of a third generation CSPG4-CAR T-cell demonstrated lasting efficacy and minimal antigen escape, at least partially due to the upregulation of CSPG4 on tumor cells by microglia-derived TNF-α in the tumor microenvironment.

While there is currently a limited number of tumor-specific antigens being targeted in GBM, this list can be expanded through the identification and analysis of tumor-specific post-translational modifications of glioma surface proteins. In particular, novel glycosylation patterns on proteins expressed by tumor cells may allow for the specific targeting of these cells, as in the case of the unique mucin 1 (MUC1) glycoepitopes that are highly expressed in a variety of cancers (171–173). Adoptively transferred T-cells stimulated against MUC1 have demonstrated promising results in clinical trials for breast and ovarian cancers (174–176). Based on the experience of MUC1, the identification and targeting of post-translational modifications of surface expressed proteins may constitute an important strategy for developing novel CAR T-cell therapies in GBM.

## Pitfalls and Opportunities for Building on Glioma Immunotherapy

### Radiographic Imaging and Pseudoprogression

In the assessment of treatment response, clinicians rely on radiographic imaging data to interpret changes in tumor size and composition (177). In particular, enhanced regions on contrast-enhanced T1-weighted images are indicative of changes to BBB permeability resulting from tumor proliferation and angiogenesis. Recognizing that the mechanisms behind immunotherapeutic response and recurrence may complicate the interpretation of radiographic information, the Immunotherapy Response Assessment in Neuro-oncology Working Group (iRANO) has proposed new guidelines to facilitate assessment of immunotherapeutic response and address the issue of pseudoprogression following immunotherapy (178). Following treatment with immunotherapy, radiographic lesions may spread beyond incipient tumor margins and include new distal and local radiographic lesions. These changes to images after immunotherapy are inherently ambiguous and may represent immune infiltration of TME, worsening tumor burden, or a mixed pathology. Radiographic pseudoprogression is transient in nature but can result in the premature termination of potentially beneficial immunotherapeutic treatments and the skewing of clinical trials toward potentially less responsive patients if left unrecognized. iRANO has proposed that clinicians consider pseudoprogression for any apparent radiographic progression within the first 6 months following the beginning of an immunotherapeutic regimen, in the absence of neurological decline, and that indications of progressive disease is confirmed only after follow-up imaging session before the patient is reclassified. Moving forward, there is a considerable need for alternative imaging techniques to be validated, such as magnetic resonance spectroscopy (MRS), perfusion and diffusion MRI, as well as PET scanning for distinguishing tumor progression from immune infiltration (179–183). In addition to improving the criteria by which radiographic images are assessed, clinicians are also encouraged to gather biopsy specimens of lesions whenever possible in order to rule out pseudoprogression and ensure that patients are given a full opportunity to benefit from immunotherapy regimens.

### Dexamethasone Administration

While genetically engineered T-cell based immunotherapy is focused upon the development of strong adaptive responses against tumor tissue in the CNS, clinical treatment of GBM often requires the administration of corticosteroids such as dexamethasone to prevent the onset of neurological symptoms associated with peritumoral edema (178). In preclinical models, dexamethasone treatment is associated with a dose-dependent decrease in lymphocyte infiltration of tumor tissue and the inhibition of T-cell maturation in the CNS by a suppressive population of monocytes (184). Furthermore, dexamethasone treatment can impede the maturation of dendritic cells and decrease their antigen presentation ability in an already immunosuppressive tumor environment. While much of these data are restricted to patients receiving high doses of corticosteroids, it is clear that the necessary administration of dexamethasone may present a substantial hurdle to some GBM patients receiving T-cell based immunotherapy unless these issues are addressed. Brown and colleagues recently addressed the question of dexamethasone in CAR T-cell therapy in a xenograft model of GBM. They found that while high-dose dexamethasone completely inhibited CAR T-cell antitumor effects, low-dose dexamethasone did not diminish antitumor effects mediated by CAR T-cell in mice (185). Dexamethasone administration will need to be considered on a patient-by-patient basis and weighed against potential and observed clinical benefit from immunotherapy. The maximum dose of dexamethasone that will not undermine therapeutic response to CAR T-cell therapy remains to be defined in the glioma setting. Ongoing and prospective CAR T-cell therapies for malignant gliomas will need to consider alternative ways to manage the symptoms of progressive disease without corticosteroids, such as through the use of the anti-angiogenesis antibody-based drug, bevacizumab. Additional methods may be required to overcome the immunosuppressive and anti-homing effects of corticosteroid treatment, including alternative delivery routes, more potent CAR T-cells, and the combined strategies for addressing immunosuppressive microenvironment that we have described.

### Lymphodepletion and Cytotoxic Therapy

Even though cyclophosphamide and fludarabine have been most widely used for lymphodepletive conditioning regimens prior to CAR T-cell therapies, we focus our discussions on a possibility for the usage of an alkylating chemotherapy agent, temozolomide (TMZ), because this is a part of the current standard-of-care alongside radiotherapy and surgical resection for patients with malignant glioma (186). As TMZ is a potent inducer of lymphopenia, it has drawn interest for use as a pre-conditioning agent before adoptive cell therapy (187–189). It is currently understood that the induction of lymphopenia is a necessary precondition for CAR T-cell therapy as it upregulates and eliminates endogenous competition for homeostatic gamma chain cytokines, such as IL-7, IL-15, and IL-2, to enhance CAR T-cell persistence (190), although lymphopenia in GBM patients treated with standard-of-care TMZ + radiation therapy did not induce compensatory upregulation of IL-7 or IL-15 (188). Suryadevara and colleagues recently used a pre-clinical mouse model of GBM treated with EGFRvIII-CAR T-cells to demonstrate that dose-intensified TMZ lymphodepletion can durably enhance CAR T-cell efficacy and persistence, while standard dose TMZ was transient and did not have significantly different effect from vehicle (189). Furthermore, they showed that dose-intensified TMZ lymphodepletion significantly increased the ratio of CAR T-cell:Treg over that with the standard dose of TMZ. Notably, TMZ and other cytotoxic therapy may be able to produce synergistic effects with CAR T-cell therapy, and there is active ongoing research to improve protection of CAR T-cells from the cytotoxic effects of these therapies (129, 191). These preclinical studies, however, need careful interpretations considering the difference in the dose and duration of therapies between humans and mice.

Conventional fractionated radiotherapy also has a profound lymphodepleting effect due to the large volume of blood that perfuses the human brain and can be affected by radiation (187, 188, 192). It has been associated with the recruitment of Tregs and MDSC, resulting in increased production of TGF-β, IL-10, and angiogenic factors in the TME (193). However, it has been hypothesized that radiotherapy might also play a positive role for CAR T-cell therapies. Radiotherapy can result in release of danger signals, such as HMGB1 and HSP70, which activate the innate and adaptive immune systems, in the context of GBM cell lines (194, 195). The cytotoxic effects of local radiotherapy also lead to the phagocytosis of tumor cells, which in turn can induce maturation of dendritic cells and enhance presentation of tumor antigens (196). In murine models, whole brain radiotherapy resulted in upregulation of MHC Class I and increased infiltration of CD8^+^ and CD4^+^ T-cells into the tumor microenvironment (197), although murine models do not allow recapitalization of fractionated radiation therapy in humans. Radiotherapy has been explored extensively (198) in combination with checkpoint blockade but relatively little in the area of CAR T-cells. However, Weiss and colleagues recently developed an NKG2D-based CAR T-cell for use in a preclinical mouse model of GBM and demonstrated improved efficacy and persistence when CAR T-cell therapy was combined with sub-therapeutic dosages of radiotherapy (199). They concluded this synergistic effect was a result of NKG2D ligands released in the TME following radiation. Importantly, while the authors did not observe any off-tumor toxicity, NKG2D ligand expression is not restricted to GBM tissue and could theoretically result in toxicity. As is the case for TMZ, careful interpretation of these preclinical studies is needed considering the relatively short duration of therapy regimens in mice.

## Conclusion

Glioma immunotherapy continues to present unique challenges due to anatomical barriers associated with the CNS and the intrinsic danger of eliciting an immune response in close proximity to neural tissue. In this review, we have discussed the most recent clinical outcomes utilizing CAR T-cells to target glioma, as well as the strategies being explored to address emerging impediments to these treatments. Limited engraftment and survival of the infused T-cells due to difficulty homing to the tumor site is a substantial problem in genetically engineered T-cell therapies for malignant glioma. Complex anatomical barriers make drainage of antigens and immune cells from the brain parenchyma into the periphery difficult and may mitigate peripheral lymphocyte activation against tumor antigens. Moreover, the homing of T-cells is limited by the BBB and an immunosuppressive TME. Altering the expression patterns of chemokines and their receptors in an effort to enhance T-cell homing to the brain tumor site have shown promise in pre-clinical studies, but these remain to be tested in the clinical setting.

In addition, the heterogeneous display of tumor antigens has resulted in tumor escape and recurrence of malignant gliomas. However, as additional tumor-associated antigens are explored for combined targeting, concerns about on-target off-tumor and systemic toxicities are warranted. Creative solutions to the combined challenges of safety and antigen heterogeneity have emerged in recent pre-clinical studies, as discussed in this review. In addition to enhancing the CAR T-cells efficacy against multiple tumor antigens, mounting evidence supports the need for combining engineered T-cells with modulators of the highly immunosuppressive TME. Recent data discussed here clearly suggest a potential for synergy of CAR T-cells with other treatments targeting the mechanisms of glioma immunosuppression. In addition to these combined strategies, engineering CAR T-cells which also express pro-survival cytokines may aid in overcoming local immunosuppression.

Several questions remain regarding the optimal delivery method and post-treatment care of GBM patients. Another challenge for clinicians designing and executing GBM clinical trials remains the administration of corticosteroids as means of avoiding the neurological symptoms of edema. The establishment of corticosteroid dosing guidelines for glioma patients receiving T-cell therapies, and the consideration of alternative interventions are likely to maximize the efficacy of CAR T-cells in the clinical setting. Despite the number of hurdles facing the use of genetically engineered T-cells for glioma immunotherapy, novel pre-clinical strategies addressing each of these hurdles continue to present opportunities for clinical progress. Creative and mindful bioengineers will need to work closely with clinical and surgical experts in order to drive forward the field of immune-oncology, both on the bench and at the bedside.

## Author Contributions

All authors contributed to the concept, review of the literature, writing, and editing the manuscript. BH contributed to drawing the figures.

### Conflict of Interest Statement

HO is an inventor of US utility patent application “H3.3 CTL peptides and uses thereof” (Case #: SF2015-163). Published research data related to this invention are discussed in this manuscript. The remaining authors declare that the research was conducted in the absence of any commercial or financial relationships that could be construed as a potential conflict of interest.
